# Immune Cell Landscape Identification Associates Intrarenal Mononuclear Phagocytes With Onset and Remission of Lupus Nephritis in NZB/W Mice

**DOI:** 10.3389/fgene.2020.577040

**Published:** 2020-11-09

**Authors:** Bin Li, Yanlai Tang, Xuhao Ni, Wei Chen

**Affiliations:** ^1^Department of Nephrology, The First Affiliated Hospital, Sun Yat-sen University, Guangzhou, China; ^2^Key Laboratory of Nephrology, National Health Commission and Guangdong Province, Guangzhou, China; ^3^Department of Pediatrics, The First Affiliated Hospital, Sun Yat-sen University, Guangzhou, China; ^4^Department of Pancreato-Biliary Surgery, The First Affiliated Hospital, Sun Yat-sen University, Guangzhou, China

**Keywords:** lupus nephritis, immune cell landscape, mononuclear phagocytes, NZB/W mice, CIBERSORT

## Abstract

**Objective:**

A challenging issue in the clinical management of lupus nephritis (LN) is the resistance to immunosuppressive therapy. We postulated that perturbed intrarenal immune cell landscape affected LN onset and remission induction, and shedding light on the characteristics of intrarenal immune cell infiltration could cultivate more efficient treatment regimens.

**Materials and Methods:**

Genome-wide expression profiles of microarray datasets were downloaded from the Gene Expression Omnibus database. The CIBERSORT algorithm was used to analyze the intrarenal immune cell landscape, followed by Pearson correlation analysis and principal component analysis. The differentially expressed genes were identified and subjected to Gene Ontology (GO) enrichment analyses and protein-protein interaction network establishment, being visualized by Cytoscape and further analyzed by CytoHubba to extract hub genes. Hub genes were also validated in the genomic dataset from kidney biopsy-proven LN patients.

**Results:**

In addition to memory B cells, monocytes and M1 macrophages were identified as two predominantly increased intrarenal immune cell types in LN-prone NZB/W mice upon nephritis onset. Most interestingly, apart from memory B cells, monocytes and M1 macrophages proportions in kidney tissue were significantly lower in early remission mice compared with late remission mice. Furthermore, GO analysis showed that intrarenal mononuclear phagocytes triggered nephritis onset mainly via the initiation of adaptive immune response and inflammatory reaction, but this functional involvement was mitigated upon remission induction. Hub genes related to LN onset in NZB/W mice were validated in the genomic dataset from kidney biopsy-proven LN patients.

**Conclusion:**

LN characterizes aberrant mononuclear phagocytes abundance and signature upon disease onset, of which the reversal is associated with early remission induction in LN-prone NZB/W mice. Mononuclear phagocytes might be an adjunctive histology marker for monitoring disease onset and stratifying LN patients in terms of response to remission induction therapy.

## Introduction

Lupus nephritis (LN) predominantly manifests as immune complex-mediated glomerular and tubulointerstitial immune complex deposition and inflammation. LN occurs in 40–60% of systemic lupus erythematosus (SLE) patients and accounts for one of the most prevalent and serious organ complications during the course of their disease (Lisnevskaia et al., 2014; Hanly et al., 2016). Introduction of corticosteroids and other immunosuppressants have profoundly contributed to improved treatment of LN. Nevertheless, a challenging issue in clinical practice is that 20% to 70% of patients diagnosed with LN are documented to be resistant to standard immunosuppressive regimens (Ginzler et al., 2005). Despite the absence of consensus denotation for a complete response after induction therapy, refractory LN is commonly denoted as failure to achieve clinical remission following proper induction immunosuppressive treatment (Chen et al., 2008). During the past decade, some clinical trials aimed to optimize therapeutic approaches by prolonging therapeutic course, increasing glucocorticoid dosage, or adding a calcineurin inhibitor ended up with varying success. In the meantime, although biomarkers for nephritis occurrence have increasingly being identified, a reliable way of predicting or determining which kind of patients will respond to induction therapy is scarce. Therefore, a more comprehensive understanding toward the mechanisms of refractory LN is demanded to generate highly specific predictive biomarkers and highly efficacious therapeutic approaches. Murine models that spontaneously develop SLE contribute a lot to our understanding of human disease and are extensively employed for the identification of effective therapeutics. Notably, the NZB/W F1 mice (F1 hybrid between New Zealand Black mouse and New Zealand White mouse, hereafter referred to as NZB/W mice) is featured by hypercellular renal impairment and fibrinoid necrosis, resembling the lesions that occur in human LN kidney biopsies. Intriguingly, induction therapy using a single dose of cyclophosphamide (CYC) administered in combination with six doses of CTLA4Ig (cytotoxic T-lymphocyte-associated protein 4 immunoglobulin G) and six doses of anti-CD154 (triple therapy) promptly reverses albuminuria and stabilizes kidney function in NZB/W mice with established nephritis (Schiffer et al., 2003), which has made it possible to dig deeper into the underlying mechanisms linked with remission induction of glomerulonephritis.

Efforts over the past decade have emphasized the critical role of innate immune cells in promoting and potentiating LN. For example, numerous studies indicate that glomerulonephritis in LN is attributable to a systemic breakdown of B cell tolerance that results in the local precipitation of immune complexes; thus, B cell targeted therapeutic strategies such as depleting B cell or blocking B cell survival factors are theoretically promising and have also been developed accordingly. Nevertheless, the efficacy of therapies targeting B cells still remains disputable (Liossis and Staveri, 2017), because several studies documented favorable outcomes in LN, while some other studies observed no clinical refinement (Melander et al., 2009; Duxbury et al., 2013). Therefore, a more thorough understanding of immune cell in the pathogenesis of LN is needed for yielding therapeutic choice with higher efficiency. Current knowledge of the subpopulations of infiltrating immune cell in LN comes mainly from the immunohistochemistry and flow cytometry studies of kidney biopsies; however, the heterogeneity of subpopulations in different disease stages remains enigmatic. Particularly, the role and mechanism of these subpopulations of infiltrating immune cell in the progress of remission induction remain unrevealed, although accumulating evidence proposes the intrarenal infiltrating immune cell as an essential factor associated with response upon immunosuppressive therapy (Melander et al., 2009; Duxbury et al., 2013; Liossis and Staveri, 2017). Hence, there is an imperative urge to unravel the immunologic mechanisms bridging immune cell state with LN progression and remission induction. These efforts may replenish key insights to precipitate better disease predictors and better-designed drugs targeting to tame LN autoimmunity.

Bioinformatics is emerging as a new interdisciplinary subject that has enabled the high-throughput and high-efficacy collection of biological information. Remarkably, the deconvolution techniques can yield surplus insight into the abundant variation of specific cell types that arise at different disease stages throughout onset, development, and treatment, thus allowing earlier and more accurate diagnosis of comorbidities and prediction of therapeutic response. For example, a newly developed bioinformatic approach, Cell-type Identification By Estimating Relative Subsets Of known RNA Transcripts (CIBERSORT) deconvolution algorithm method^1^, has been successfully applied to assess the levels of 22 kinds of immune cell types in large amounts of heterogeneous samples based on gene expression profiles, allowing large-scale interpretation of mRNA compound for defining novel cellular biomarkers and therapeutic targets. On the other hand, even though several recent studies have contributed to defining the immune cell infiltration state milieu of LN by integrated bioinformatic analyses (Arazi et al., 2019; Cao et al., 2019), transcriptional signatures of infiltrating immune cell landscape that distinguish LN subgroups with the varied response to remission induction have not yet been described.

We hypothesized that intrarenal immune infiltration state possibly has predictive value in delineating the response to therapy. Thus, to clarify the association between immune infiltration and LN onset as well as remission induction, the microarray datasets of LN-prone NZB/W mice at various disease stages in Gene Expression Omnibus (GEO) were dissected by a variety of bioinformatics techniques including CIBERSORT to define distinct subgroups. Findings from this study correlate LN onset with exuberant mononuclear phagocytes abundance and signature, whose reversal is associated with early remission induction. This explorative study extends our knowledge about mononuclear phagocytes as a future platform for diagnosis and precision medicine in LN.

## Materials and Methods

### Overview of Microarray Datasets Collection

**FIGURE 1:**
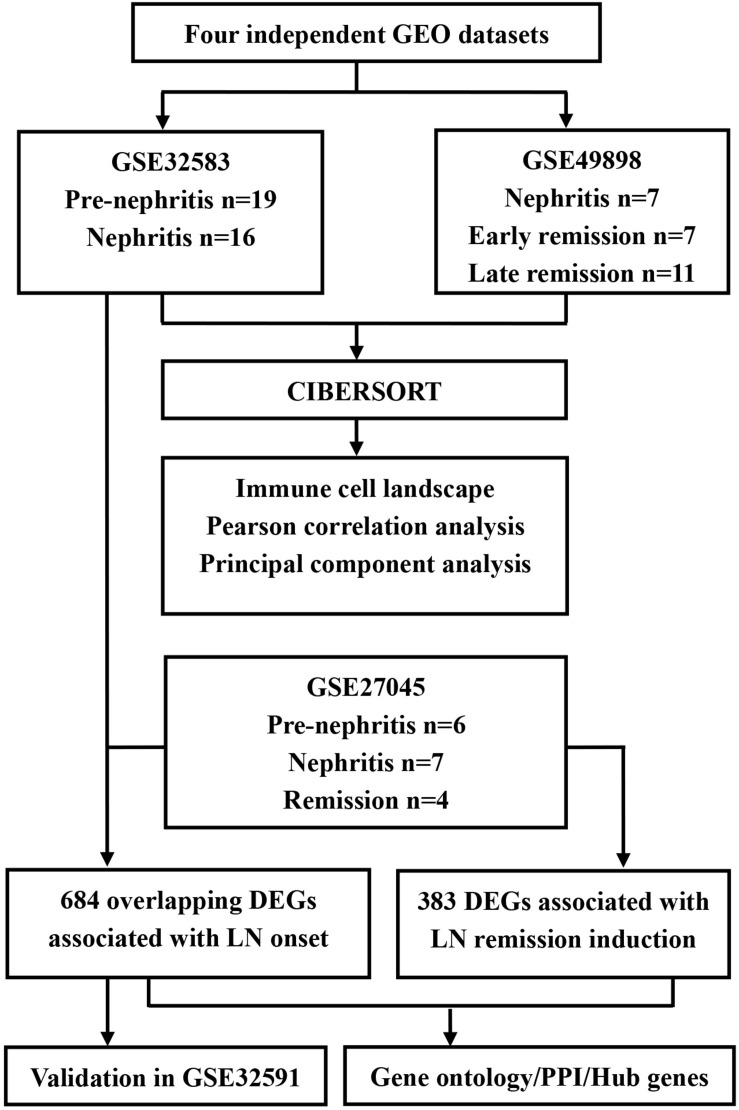
Flowchart of the analyses used in this study. GEO, Genome Expression Omnibus; DEGs, differentially expressed genes; PPI, protein-protein interaction.

The diagram of the overall study design and analysis process is displayed in Figure 1. We screened the qualified datasets that contained comprehensive intrarenal gene-expression profiles of kidneys from SLE-prone murine models or human LN patients, because these dataset types can help determine intrarenal immune cell landscape at different disease stages of LN. As a result, four independent LN gene expression profiles (GSE32583, GSE49898, GSE27045, and GSE32591) were downloaded from the GEO database and exploited to identify or validate differentially expressed genes (DEGs). Supplementary Table S1 provides additional information about all of the above four datasets. A detailed description of murine and human RNA extraction, microarray preparation and processing, as well as gene-expression data processing and analysis could be retrieved from the corresponding original literature (Schiffer et al., 2003; Reddy et al., 2008; Bethunaickan et al., 2011, 2014; Berthier et al., 2012).

In GSE32583 dataset, the NZB/W mice were allocated into pre-nephritis control group (without proteinuria) and nephritis group (proteinuria > 300 mg/dl). Therefore, GSE32583 was used to discover and compare intrarenal gene expression between pre-nephritis and nephritis mice. The expression matrix of 19 pre-nephritis mice (GSM807484–GSM807502) and 16 nephritis mice (GSM807503–GSM807518) were obtained to profile the infiltrating immune cell (Schiffer et al., 2003; Berthier et al., 2012).

In GSE49898 dataset, nephritic NZB/W mice (proteinuria > 300 mg/dl) were treated with a single dose of CYC and 6 doses of CTLA4Ig and anti-CD154. Mice that attained proteinuria ≤ 30 mg/dl within 3–4 weeks post induction treatment fell into the early remission group and showed complete histologic remission. By contrast, mice that obtained proteinuria ≤ 30 mg/dl more than 5–14 weeks post induction treatment fell into the late remission group and displayed only partial histologic remission by light microscopy (Chan et al., 1997). Therefore, GSE49898 was employed to compare the intrarenal gene expression among nephritis, early remission, and late remission NZB/W mice. After data processing, the expression matrix of 7 nephritis mice (GSM1209137–GSM1209143), 7 early remission mice (GSM1209145–GSM1209151), and 11 late remission mice (GSM1209152–GSM1209162) were obtained to profile the infiltrating immune cell (Bethunaickan et al., 2014).

Similarly, in GSE27045, nephritic NZB/W mice were treated with a single dose of CYC and 6 doses of CTLA4Ig and anti-CD154 if proteinuria > 300 mg/dl occurred. Remission was defined as proteinuria ≤ 30 mg/dl, and some young NZB/W mice were allocated into pre-nephritis group. The F4/80^hi^ (a classic mononuclear phagocyte marker) mononuclear phagocytes, which acquire an activated phenotype during active nephritis and reverse upon remission induction, were sorted and isolated by flow cytometry from single-cell suspensions of perfused kidneys. Therefore, GSE27045 was utilized to compare the gene expression of F4/80^hi^ intrarenal mononuclear phagocytes among pre-nephritis, nephritis, and remission NZB/W mice. The expression matrix of 6 pre-nephritis mice (GSM667532–GSM667537), 7 nephritis mice (GSM667538–GSM667544) and 4 remission mice (GSM667545–GSM667548) were obtained for defining mononuclear phagocytes-derived genes associated with LN onset and remission induction (Bethunaickan et al., 2011).

In GSE32591, a total of 47 renal biopsies from the European Renal cDNA Bank (Schmid et al., 2006) were collected according to the guidelines of the respective local ethics committees. The demographic, clinical, and histologic characteristics of the included patients could be retrieved from the original literature (Berthier et al., 2012). Therefore, GSE32591 was exploited to investigate and compare the gene expression of glomeruli and tubulointerstitial compartments of renal biopsies from LN patients (*n* = 32) and pretransplant healthy living donors (*n* = 15). The expression matrix of renal tubulointerstitial compartment including 32 LN patients (GSM807842–GSM807873) and 15 healthy living donors (GSM807874–GSM807888), as well as renal glomeruli compartment including 32 LN patients (GSM807889–GSM807920) and 14 healthy living donors (GSM807921–GSM807934), were obtained for validation analyses in our current study (Berthier et al., 2012).

### Evaluation of Immune Cell Infiltration by CIBERSORT Analyses

To determine the immune cell landscape in kidney tissues, the analytical platform CIBERSORT (see footnote 1) with the reference of 1000 permutations and LM22 signature was employed. The CIBERSORT deconvolution algorithm has been validated to accurately and reliably calculate 22 types of immune cell fractions dependent on microarray expression data. These immune cells are composed of naive B cells, memory B cells, plasma cells, CD8+ T cells, naive CD4+ T cells, resting memory CD4+ T cells, activated memory CD4+ T cells, follicular helper T cells, regulatory T cells (Tregs), gamma delta T cells, resting NK cells, activated NK cells, monocytes, M0 macrophages, M1 macrophages, M2 macrophages, resting dendritic cells, activated dendritic cells, resting mast cells, activated mast cells, eosinophils, and neutrophils. The significant alteration of immune cell fractions was recognized according to the threshold of the Wilcoxon test at *p*-value < 0.05. Associations between different immune cell subtypes were evaluated via Pearson correlation coefficient.

### Principal Component Analysis

Principal component analysis (PCA) is often used as a technique in exploratory data analysis for variable dimensionality reduction. Therefore, PCA was utilized in the current study to ascertain primary sources of variance in the fraction of diverse infiltrating immune cell types among different groups, and the prominent sources of variance can likely be the diagnostic clues for LN onset or predictive biomarkers for early LN remission induction. To be specific, log-ratio PCA is proposed as an efficient tool for the exploration of compositional data (Graffelman et al., 2019); thus, we followed that approach by applying the centered log-ratio (*clr*) transformation to the compositional data. In brief, the compositional data of immune cell fractions derived from CIBERSORT was expressed in isometric coordinates. Afterward, PCA was performed to decompose the normalized, log10-transformed immune cell composition matrix by using the dudi.pca function in R. Resulting loadings and scores were back-transformed to the *clr* space where the compositional biplot could be shown.

### Identification of DEGs and Functional Enrichment Analyses

An R-based web application, GEO2R, was employed to obtain DEGs in GSE datasets by comparing the expression values among different subgroups and using the GEOquery and the linear models for microarray data (LIMMA) package of R. The adjusted *p*-values (calculated by Benjamini and Hochberg false discovery rate method) via GEO2R tool were adopted to avoid the occurrence of false-positive results. DEGs (adjusted *p*-value < 0.05) between pre-nephritis group and nephritis group mice were identified in GSE32583 and GSE27045 dataset, respectively. GSE27045 dataset contained the genomic profile of kidney-isolated F4/80^hi^ mononuclear phagocytes, hence the overlapping DGEs (adjusted *p*-value < 0.05) between GSE27045 and GSE32583 datasets were further identified to define mononuclear phagocytes-specific DEGs associated with LN onset. On the other hand, DEGs (adjusted *p*-value < 0.05) between nephritis and remission group mice were identified in GSE49898 and GSE27045 dataset, respectively. However, statistical analyses showed that there were no DEGs (adjusted *p*-value < 0.05) between nephritis and early remission NZB/W mice in GSE49898 dataset, hence F4/80^hi^ mononuclear phagocytes-specific DEGs (adjusted *p*-value < 0.05, and |log2 fold change (FC)| > 1) between nephritis and remission NZB/W mice in GSE27045 dataset were further pinpointed and deemed as mononuclear phagocytes-derived genes associated with LN remission induction. These DEGs or overlapping DEGs were included for Gene Ontology (GO) enrichment analyses through WebGestalt (WEB-based Gene SeT AnaLysis Toolkit), and Ggplot2 of R was applied to draw heatmap for visualization of the overlapping DEGs. Furthermore, protein-protein interaction networks of the overlapping DEGs were established via STRING (Search Tool for the Retrieval of Interacting Genes database) online tool and visualized in Cytoscape software. Hub genes with a high degree of connectivity were extracted by applying the plug-in of CytoHubba. LN onset-related hub genes were also validated in GSE32591 dataset that included kidney biopsies from LN patients.

## Results

### Composition of Immune Cell Between Pre-nephritis and Nephritis NZB/W Mice by CIBERSORT

**FIGURE 2:**
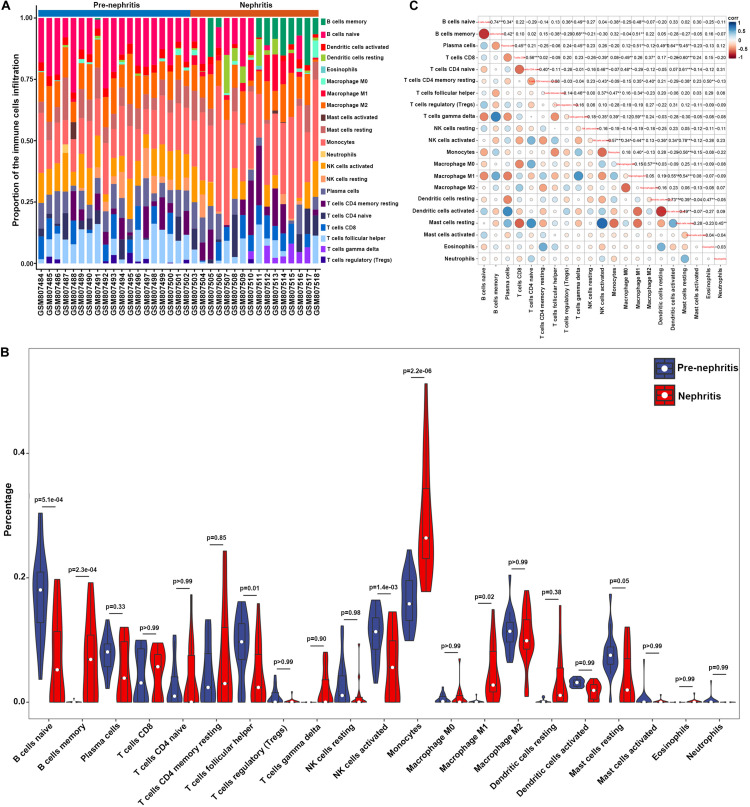
Composition of infiltrating immune cell subpopulations in kidney tissues from pre-nephritis and nephritis NZB/W mice in GSE32583 dataset. **(A)** The fraction of infiltrating immune cell subpopulations was determined by CIBERSORT. **(B)** Comparison of renal immune infiltration between pre-nephritis and nephritis NZB/W mice. **(C)** Correlation among infiltrating immune cell subpopulations.

To determine whether renal infiltrating immune cell corresponded with nephritis onset, CIBERSORT was utilized to quantify the immune cell proportions within kidney samples (GSE32583 dataset). Compared to pre-nephritis mice, nephritis NZB/W mice were characterized by obviously lower proportions in naïve B cells, follicular helper T cells, and activated NK cells, but notably higher proportions in memory B cells, monocytes, and M1 macrophages (Figures 2A,B and Supplementary Table S2). Particularly, monocytes accounted for the highest proportion and the most pronounced elevation among all the immune cell types in the nephritic kidney from NZB/W mice (Figures 2A,B). This result is in line with a very recent bioinformatic study that recognized monocytes as the most significantly increased and the most abundant infiltrating immune cell type in kidney biopsy from human LN subjects (Cao et al., 2019). Furthermore, a significantly positive correlation between monocytes and M1 macrophages (*r* = 0.40) was presented by the correlation analyses (Figure 2C). Intriguingly, both the percentages of monocytes and M1 macrophages were significantly negatively correlated with naïve B cells (*r* = −0.38 and −0.48 respectively), follicular helper T cells (*r* = −0.47 and −0.34 respectively), activated NK cells (*r* = −0.57 and −0.44 respectively), and resting mast cells (*r* = −0.56 and −0.54 respectively), but significantly positively correlated with gamma delta T cells (*r* = 0.39 and 0.59 respectively) (Figure 2C). The broadly notable correlations between monocytes/M1 macrophages and other immune cell types underpin the critical role of mononuclear phagocytes in orchestrating LN occurrence.

**FIGURE 3:**
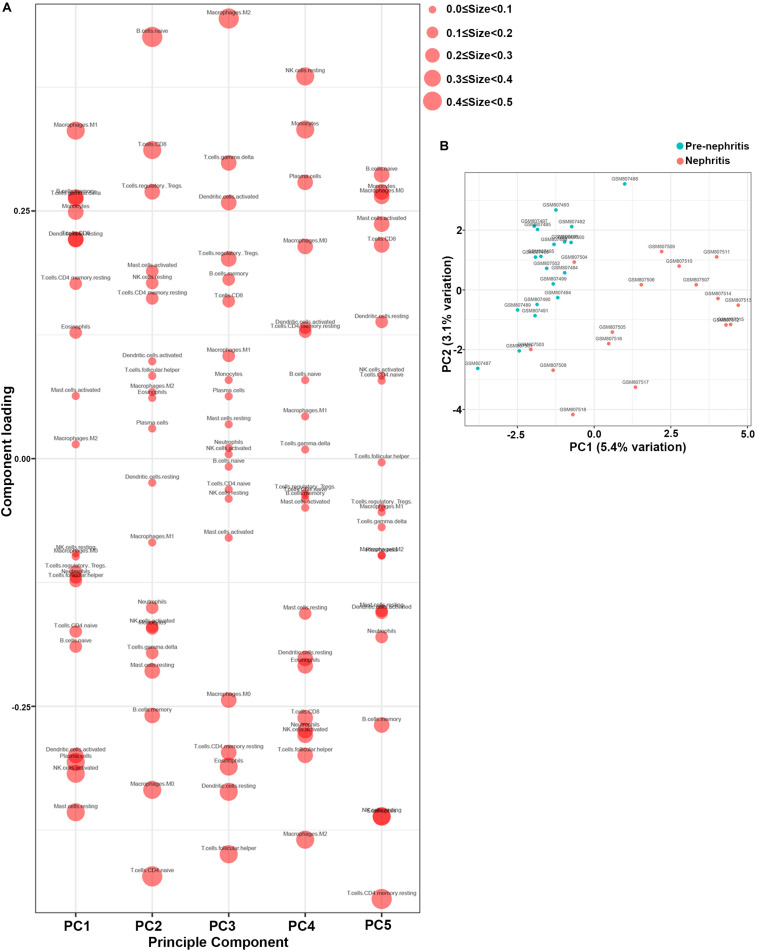
Principal component analysis (PCA) was performed to reveal differences in immune cell landscape between pre-nephritis and nephritis NZB/W mice in GSE32583 dataset. **(A)** Component loading in PCA results. **(B)** Score plot for PC1 and PC2. The percentages of variance explained by PC1 and PC2 are in the axis labels. PC, principal component.

PCA was subsequently performed to evaluate if the fractions of infiltrating immune cell could be exploited to distinguish the diagnosis of LN onset. M1 macrophages, memory B cells, gamma delta T cells, and monocytes were found to be the major components of principal component (PC) 1, and they were positively associated with LN onset (Figure 3A). Nevertheless, data visualization by PCA in Figure 3B shows that the first two PCs explained only 8.5% of the variance, indicating the mere composition of immune cells in LN kidney tissue was insufficient to discriminate pre-nephritis from nephritis mice. Taken together, the above results suggested aberrant immune infiltration in LN kidney tissues as a tightly regulated process that influenced the pathogenesis of LN onset. Specifically, it is worth noting that mononuclear phagocytes including monocytes and M1 macrophages were dramatically augmented in the nephritic kidney from NZB/W mice, making the mononuclear phagocytes abundance a potential adjuvant diagnostic biomarker for LN occurrence.

### Composition of Immune Cell Between Early Remission and Late Remission NZB/W Mice by CIBERSORT

**FIGURE 4:**
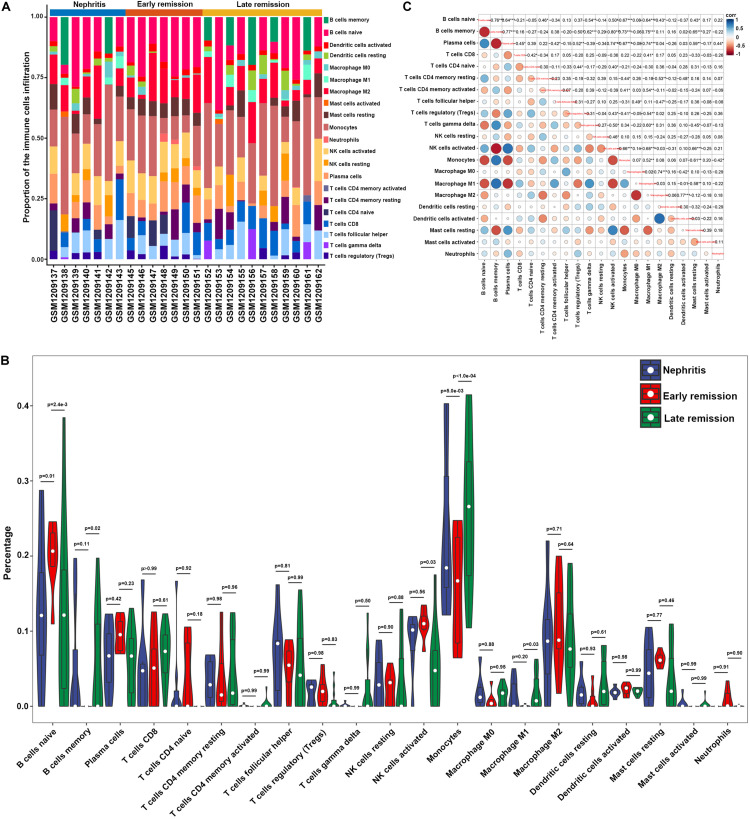
Composition of infiltrating immune cell subpopulations in kidney tissues from nephritis, early remission, and late remission NZB/W mice in GSE49898 dataset. **(A)** The fraction of infiltrating immune cell subpopulations was determined by CIBERSORT. **(B)** Comparison of renal immune infiltration among nephritis, early remission, and late remission NZB/W mice. **(C)** Correlation among infiltrating immune cell subpopulations.

In order to examine whether the renal infiltrating immune cells affected nephritis remission upon immunosuppressive therapy, CIBERSORT was further utilized to profile the immune cell landscape within kidney samples from LN mice at different disease stages (GSE49898 dataset). Consistent with the result from CIBERSORT analyses in GSE32583 (Figures 2A,B), monocytes still accounted for the highest proportion among all the immune cells in nephritic kidney (Figures 4A,B and Supplementary Table S3). More importantly, in comparison with late remission mice, early remission mice attained noticeably higher proportions of naïve B cells, activated NK cells, but significantly lower proportions of memory B cells, monocytes, and M1 macrophages (Figures 4A,B and Supplementary Table S3). Despite the suppressed memory B cells, the notably decreased percentages of mononuclear phagocytes including monocytes and M1 macrophages in early remission kidney samples indicated an essential role of attenuating mononuclear phagocytes abundance in contributing to early response upon immunosuppressive therapy. Besides, the positive correlation between monocytes and M1 macrophages was also present in the GSE49898 dataset (Figure 4C), consistent with the results from GSE32583 as mentioned earlier. The percentages of monocytes and M1 macrophages were found negatively correlated with naïve B cells (*r* = −0.67 and −0.64 respectively), plasma cells (*r* = −0.67 and −0.74 respectively), regulatory T cells (Tregs) (*r* = −0.41 and −0.54 respectively), activated NK cells (*r* = −0.66 and −0.68 respectively), resting mast cells (*r* = −0.61 and −0.58 respectively), but positively correlated with memory B cells (*r* = 0.73 and 0.75 respectively) and activated memory CD4 T cells (*r* = 0.41 and 0.54 respectively) (Figure 4C). The positive correlation between mononuclear phagocytes and B or T cells was in line with previous evidence showing the full capacity of mononuclear phagocytes to facilitate B and T cell responses and orchestrate adaptive autoimmune response (Gkirtzimanaki et al., 2018).

**FIGURE 5:**
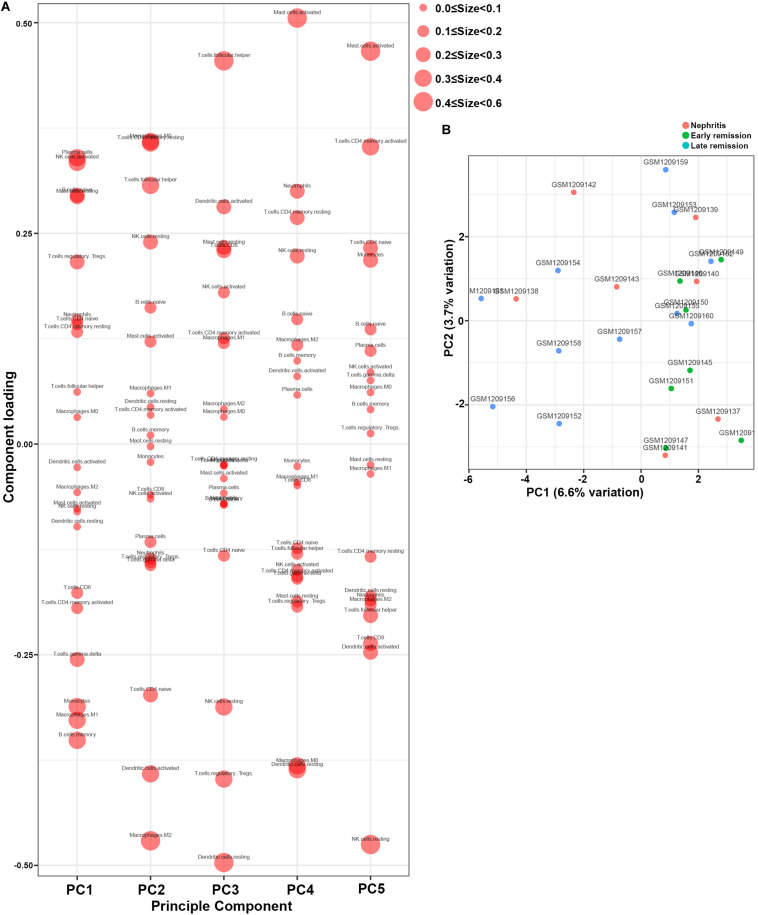
Principal component analysis (PCA) was applied to reveal differences of immune cell landscape among nephritis, early remission, and late remission NZB/W mice in GSE49898 dataset. **(A)** Component loading in PCA results. **(B)** Score plot for PC1 and PC2. The percentages of variance explained by PC1 and PC2 are in the axis labels. PC, principal component.

Furthermore, PCA results demonstrated that memory B cells, M1 macrophages, monocytes, and gamma delta T cells were the major components of PC1 that were negatively associated with remission induction upon immunosuppressive therapy (Figure 5A). These results coordinated with findings in Figure 3A to suggest those four immune cell types as the major components that contributed to LN onset and hampered early remission induction. However, the first two PCs only explained 10.3% variation (Figure 5B), suggesting that the mere composition of immune cells in LN kidney tissue was not compelling to distinguish early remission mice from nephritis or late remission mice. Collectively, the above results implied that dysregulated immune infiltration in LN might convey important meanings for predicting the response to immunosuppressive therapy. Particular attention should be paid that the fraction of mononuclear phagocytes including monocytes and M1 macrophages were significantly lower in early remission LN mice, indicating the possibility of proposing mononuclear phagocytes abundance as a predictive marker for discovering potential refractory LN patients.

### GO Analyses of Mononuclear Phagocytes-Specific DEGs Associated With LN Onset

Considering the notable amplification of mononuclear phagocytes abundance upon nephritis onset, we sought to examine the functional involvement of mononuclear phagocytes in the development of LN through bioinformatic analyses. Interestingly, GSE27045 encompassed the microarray data from NZB/W mice kidney-isolated F4/80^hi^ (a broadly accepted mononuclear phagocyte marker) mononuclear phagocytes (Bethunaickan et al., 2011; Waddell et al., 2018), which were the dominant intrarenal origin of proinflammatory cytokines and chemokines. These F4/80^hi^ mononuclear phagocytes acquired an activated phenotype during active nephritis and reversed upon remission induction (Bethunaickan et al., 2011). Hence we assumed that the overlapping DEGs between GSE27045 and GSE32583 could represent the mononuclear phagocytes-specific genes closely associated with LN onset. Accordingly, 684 overlapping DEGs (adjusted *p*-value < 0.05) between GSE27045 and GSE32853 (Supplementary Table S4) were identified for subsequent functional enrichment analysis.

**FIGURE 6:**
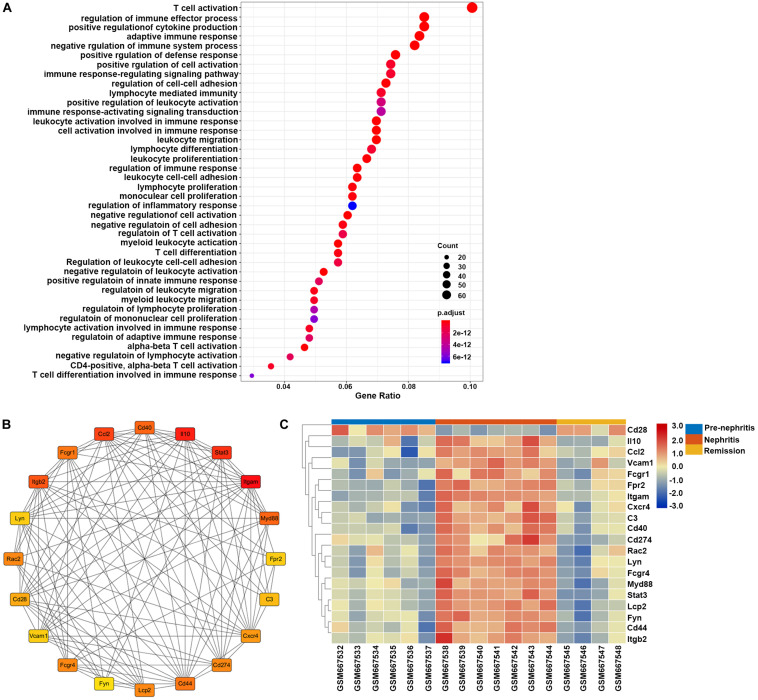
Gene set enrichment analysis of the overlapping DEGs between GSE27045 and GSE32583, which were deemed as genes linked with LN onset in NZB/W mice. **(A)** GO enrichment analysis of overlapping DEGs between GSE27045 and GSE32583. The *y*-axis labels represent clustered GO terms, and the Gene Ratio represents the ratio of the number of genes enriched in one GO term to the number of DEGs. **(B)** Network analysis of identified hub genes from overlapping DEGs between GSE27045 and GSE32583. **(C)** Heatmap of identified hub genes from overlapping DEGs between GSE27045 and GSE32583. GO, gene ontology; DEGs, differentially expressed genes.

GO enrichment analysis found that the overlapping DEGs between GSE27045 and GSE32583 were mainly enriched in the mobilization of adaptive immune response and proinflammatory reaction, with the top three enriched GO terms being T cell activation, regulation of immune effector process, and positive regulation of cytokine production (Figure 6A). This result underpinned the vital role of mononuclear phagocytes signature in mobilizing the intrarenal adaptive immune response, thus cultivating the inflammatory reaction during the development of LN onset. In addition, 20 hub genes were extracted from a constructed protein-protein interaction network established by these overlapping DEGs (Figure 6B and Supplementary Table S5). Heatmap showed that most of these hub genes were markedly increased in nephritic kidney samples, except Cd28 that were downregulated (Figure 6C). In particular, dramatically increased hub genes like CD40 (cluster of differentiation 40), Itgam (integrin subunit alpha M), C3 (complement 3), and Myd88 (myeloid differentiation primary response 88) have previously been proven to play essential roles in the expansion and activation of B and T cells (Zarnegar et al., 2004; Quigley et al., 2009; Griffin and Rothstein, 2011; Liszewski et al., 2013), supporting the role of mononuclear phagocytes signature in bridging innate immune response with the adaptive autoimmune response in the context of LN. Meanwhile, elevated hub genes like Ccl2 (C-C motif ligand 2), Cxcr4 (C-X-C chemokine receptor type 4), Il10 (interleukin 10), and Vcam1 (vascular cell adhesion molecule 1) have already been proven to play critical roles in recruiting immune cells for triggering and driving inflammation (Ishida et al., 1994; Daly and Rollins, 2003; Kong et al., 2018; Garcia-Cuesta et al., 2019), strengthening the notion of targeting mononuclear phagocytes signature to ameliorate renal pathology in the context of LN.

### GO Analyses of Mononuclear Phagocytes-Specific DEGs Associated With LN Remission Induction

Although we postulated that the overlapping DEGs between GSE27045 and GSE49898 could be ascertained and acknowledged as the potential intrarenal mononuclear phagocytes-specific DEGs highly associated with LN remission induction, statistical analysis found no DEGs (adjusted *p*-value < 0.05) between nephritis and early remission group in GSE49898. Therefore, 383 intrarenal F4/80^hi^ mononuclear phagocytes-derived DEGs (adjusted *p*-value < 0.05, and |log2 fold change (FC)| > 1, Supplementary Table S6) between nephritis and remission NZB/W mice were directly recognized and deemed as mononuclear phagocytes-specific and LN remission induction-related genes for the following functional enrichment analysis.

**FIGURE 7:**
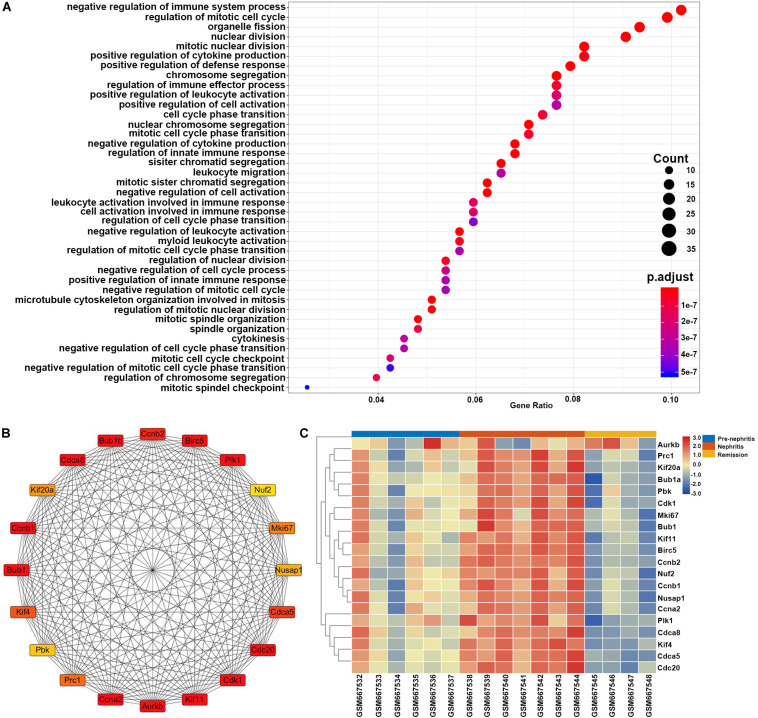
Gene set enrichment analysis of the LN remission-related DEGs in NZB/W mice from GSE27045. The *y*-axis labels represent clustered GO terms, and the Gene Ratio represents the ratio of the number of genes enriched in one GO term to the number of DEGs. **(A)** GO analysis of LN remission-associated DEGs in GSE27045. **(B)** Network analysis of identified hub genes from LN remission-associated DEGs in GSE27045. **(C)** Heatmap of identified hub genes from LN remission-associated DEGs in GSE27045.

GO enrichment analysis found that the top enriched GO term of these DEGs was negative regulation of immune system process, which indicated that immunosuppressive therapy initiated the functional signature in mononuclear phagocytes to counteract autoimmune response with LN kidney (Figure 7A). This result together with the GO enrichment analysis of overlapping DEGs between GSE27045 and GSE32583 suggested that mononuclear phagocytes-mediated immune response and inflammatory reaction were eased upon immunosuppressive therapy in NZB/W mice. This reversal possibly played the dominant role in achieving remission induction, in agreement with the previous consensus that successfully induced remission was linked with reversal of renal autoimmunity and inflammation signature. Furthermore, heatmap of 20 hub genes extracted from DEGs between nephritis and remission mice in GSE27045 (Figure 7B and Supplementary Table S7) demonstrated that almost all these hub genes were markedly elevated in the nephritic kidney, but significantly reversed or downregulated upon remission induction (Figure 7C). This result highlighted the potential role of these critical genes in impeding remission induction; despite how these hub genes interfere the response to immunosuppressive regimens in the context of LN remains elusive. Intriguingly, in line with our findings, some previous lines of evidence have already shown that some of these hub genes like Cdk1 (cyclin-dependent kinase 1) (Wu et al., 2016) and Mki67 (Rahbar et al., 2018) were tightly involved in the pathogenesis of SLE or LN.

### Validation of LN Onset-Related Hub Genes in Human Kidney Biopsies From LN Patients

**FIGURE 8:**
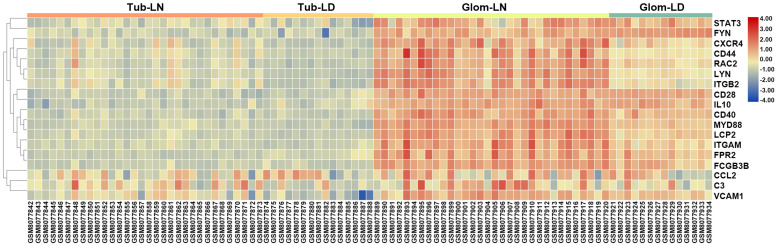
Validation of LN onset-related hub genes from overlapping DEGs between GSE27045 and GSE32583 in the GSE32591 dataset. A heatmap of LN onset-related hub genes identified from the overlapping DEGs between GSE27045 and GSE32583 in the GSE32591 genomic dataset which included kidney biopsies from LN patients. Tub, tubulointerstitial; LN, lupus nephritis; LD, healthy living donor; Glom, glomeruli.

To determine the relevance to human disease, LN onset-related hub genes identified from overlapping DEGs between GSE27045 and GSE23852 were further validated in kidney biopsies from LN subjects in GSE32591. Results demonstrated that these hub genes were apparently upregulated in glomeruli instead of tubulointerstitial compartments, indicating the functional difference of mononuclear phagocytes in these two intrarenal microenvironments. Moreover, of the total 20 mouse LN onset-related hub genes (corresponding to 18 human genes), most demonstrated a striking level of concordance as that in LN-prone NZB/W mice, as evidenced by notably higher expression level in glomeruli from LN patients compared to healthy living donors. The top three dramatically upregulated hub genes were ITGB2 (integrin subunit beta 2), LYN (LYN proto-oncogene, Src family tyrosine kinase), and CXCR4 (C-X-C motif chemokine receptor 4) (Figure 8 and Supplementary Table S8). It is worth noting that the hub gene CXCR4 was previously found abundant in kidney biopsies from SLE patients, and CXCR4 antagonist administration could improve disease severity and nephritis in murine lupus models (Chong and Mohan, 2009). Therefore, this overlap of a substantial subset of molecular markers with those in human LN kidneys highlighted mononuclear phagocytes signature as a cross-species shared feature (Olaru et al., 2018).

## Discussion

High frequency of being resistant to immunosuppressive therapy contributes to adverse renal outcomes in LN (Ginzler et al., 2005). Only 50–70% of LN patients achieved remission by the current therapeutic regimens, and progression to end-stage renal disease still occurs to 10–20% patients over 5–10 years (Tektonidou et al., 2016). Recent advances have pinpointed the involvement of a diverse range of immune cells in LN progression, despite the fact that the underlying pathophysiology is not fully understood. In the current study, through the in-depth bioinformatic analyses of LN transcriptomics data, we implemented a comprehensive deconstruction of intrarenal immune cell landscape in LN-prone NZB/W mice at different disease stages. In consequence, our findings highlighted that amplified mononuclear phagocytes abundance and signature were attributable to not only disease onset but also the failure of early remission induction. Besides, functional enrichment analyses delineated the functional profile of mononuclear phagocytes in driving nephritis onset and hampering the early remission induction. Collectively, our research revealed the characteristic of mononuclear phagocytes abundance and signature to segregate pre-nephritis mice from nephritis mice, as well as early remission mice from late remission mice. These findings suggest the promise of utilizing mononuclear phagocytes as prognostic and predictive biomarkers, as well as potential therapeutic targets for LN administration.

Abnormalities in mononuclear phagocytes phenotype, function, and activation are increasingly being associated with the pathogenesis of autoimmune diseases including SLE (Zhang et al., 2016; Burbano et al., 2018) and rheumatoid arthritis (Ma et al., 2019), despite that the underlying regulatory mechanism of mononuclear phagocytes in the context of LN has not been fully elucidated. Although the strong correlation of mononuclear phagocytes with disease activity or organ damage in LN has been recognized for over a decade, only recently has the research been directed toward the understanding of the cellular and molecular mechanism. For example, LN subjects with severer forms (Class III and Class IV) or LN-prone murine models with severer histological impairment displayed more significant intrarenal monocytes infiltration (Bergtold et al., 2006; Yoshimoto et al., 2007; Menke et al., 2011; Bignon et al., 2014; Barrera Garcia et al., 2016); however, only lately have several lines of evidence shown that three different LN-prone murine models and LN patients were all characterized by the glomeruli-specific accumulation of monocytes with a unique capacity to trigger early immune complex-induced inflammation (Olaru et al., 2018; Kuriakose et al., 2019). Meanwhile, activated renal macrophage has been acknowledged as the hallmark of LN onset and failed remission induction in both LN-prone murine models and human LN subjects (Schiffer et al., 2008; Menke et al., 2009; Triantafyllopoulou et al., 2010; Olmes et al., 2016; Kim et al., 2020), while blockade of macrophage infiltration ameliorated renal inflammation and proteinuria in LN-prone murine models (Kishimoto et al., 2018; Luan et al., 2019).

The failure of most clinical trials of rationally designed therapies in both SLE and LN pleads for an imperative need to dissect the potential mechanisms that impel LN (Arazi et al., 2019). Interpreting the relevance of mononuclear phagocytes in LN and the parallel mechanisms could drive the future identification of more potent therapeutic strategies. Over the last decade, newly emerging technologies like omics-based techniques (e.g., genomics, transcriptomics, and proteomics) offer a promising path toward this goal by expanding our understanding of the molecular basis of LN. Furthermore, multiple computational algorithms like CIBERSORT enable the direct enumeration of immune cell subsets linked with LN kidney conditions. Consistent with previous preclinical and clinical evidence, our current study conducted by comprehensive bioinformatic analyses for the first time depicted that mononuclear phagocytes abundance and signature could be a robust biological marker of LN progression and predictor of failed early remission induction. More importantly, functional enrichment analyses further strengthened the essential role of mononuclear phagocytes in triggering adaptive immune response and inflammation within the kidney upon LN onset, which was however substantially quenched after initiation of immunosuppressive therapy.

Besides, correlation analyses together with GO functional analyses indicated that mononuclear phagocytes signature and other immune cell signals were interwound. This crosstalk orchestrated the adaptive immunity like the differentiation of monocyte to macrophage with increased capacity to drive B and T cell response. For example, findings from Pearson correlation analyses verified the close correlation between the fractions of intrarenal mononuclear phagocytes and the proportions of B cells and T cells. This result was consistent with GO functional enrichment analyses results showing that LN onset-related DEGs in intrarenal mononuclear phagocytes were intensively enriched in lymphocyte differentiation, proliferation, and activation. Last but not least, the majority of the LN onset-associated hub genes identified in NZB/W mice were validated in human LN kidney genomic profile. These validating hub genes demonstrated a similar elevating trend in the glomerular compartment of LN patients, indicating the shared common and unique features between LN-prone murine model and human LN.

## Conclusion

In the current study, multiple computational approaches were performed to deconvolve the bulk transcriptome data from whole kidney tissue into immune cell type-specific fractions. These results delineated the intrarenal immune cell landscape and estimated the percentages alterations associated with LN onset and remission induction in NZB/W mice. Specifically, our findings identify the significantly amplified mononuclear phagocytes abundance and signature as the source of biological markers that forecast LN onset and retarded remission induction. These discoveries may be extremely pivotal for clinical trial designs and management of novel immunosuppressive therapies in patients with different remission period by shedding light on the suitability of combining mononuclear phagocytes-targeted adjuvant regimen against LN.

## Data Availability Statement

The LN-related microarray datasets GSE32583, GSE49898, GSE 27045, and GSE32591 were downloaded from the GEO database (https://www-ncbi-nlm-nih-gov.eproxy.lib.hku.hk/gds).

## Author Contributions

BL and WC designed this study. BL, YT, and XN retrieved and analyzed the data. BL, YT, and XN drafted the manuscript. WC edited and revised the manuscript. All authors read and approved the final manuscript.

## Conflict of Interest

The authors declare that the research was conducted in the absence of any commercial or financial relationships that could be construed as a potential conflict of interest.

## Footnotes

1 https://cibersort.stanford.edu/

## Supplementary Material

Supplementary Table 1 Characteristic of all the datasets used in this study.

Supplementary Table 2 CIBERSORT fraction of infiltrating immune cells in GSE32583.

Supplementary Table 3 CIBERSORT fraction of infiltrating immune cells in GSE49898.

Supplementary Table 4 LN onset-related DEGs overlapped between GSE27045 and GSE32583.

Supplementary Table 5 LN onset-related hub genes identified from DEGs overlapped between GSE27045 and GSE32583.

Supplementary Table 6 LN remission-related DEGs in GSE27045.

Supplementary Table 7 LN remission-related hub genes in GSE27045.

Supplementary Table 8 Validation of LN onset-related hub genes in GSE32591.
