# Supplementary material for: Immune Cell Landscape Identification Associates Intrarenal Mononuclear Phagocytes With Onset and Remission of Lupus Nephritis in NZB/W Mice
Source: Front Genet. 2020 Nov 9;11:577040. doi: 10.3389/fgene.2020.577040 (PMC7693546; doi:10.3389/fgene.2020.577040)
Supplement: Supplementary Table 1 — Characteristic of all the datasets used in this study. [file Table_1.DOCX]

**Supplementary Table 1 ∣Profiling datasets of Gene Expression Omnibus (GEO).**

| **GEO ID** | **Species** | **Strain** | **Tissue or Cell Source** | **Overall Design** | **Groups Definition** | **Platform** | **Year** | **Country** | **Reference** |
| --- | --- | --- | --- | --- | --- | --- | --- | --- | --- |
| GSE32583 | Mouse | NZB/W | Kidney | RNA from whole kidneys was extracted and processed for hybridization on Affymetrix microarrays. | (1) Pre-nephritis (n=19, GSM807484-GSM807502)  (2) Nephritis (n=16, GSM807503-GSM807518) | GPL7546, Affymetrix GeneChip Mouse Genome 430 2.0 Array [CDF: Mm_ENTREZG_10] | 2012 | USA | (Berthier et al., 2012; Schiffer et al., 2003) |
| GSE49898 | Mouse | NZB/W | Kidney | RNA from whole kidneys was extracted and processed for hybridization on Affymetrix microarrays. | (1) Nephritis (n=7, GSM1209137-GSM1209143)  (2) Eearly remission (n=7, GSM1209145-GSM1209151)  (3) Late remission (n=11, GSM1209152-GSM1209162) | GPL7546, Affymetrix GeneChip Mouse Genome 430 2.0 Array [CDF: Mm_ENTREZG_10] | 2014 | USA | (Bethunaickan et al., 2014) |
| GSE27045 | Mouse | NZB/W | Kidney F4/80^hi^ mononuclear phagocyte | RNA from intrarenal mononuclear phagocyte was extracted and processed for hybridization on Affymetrix microarrays. | (1) Pre-nephritis (n=6, GSM667532-GSM667537)  (2) Nephritis (n=7, GSM667538-GSM667544)  (3) Remission (n=4, GSM667545-GSM667548) | GPL7546, Affymetrix GeneChip Mouse Genome 430 2.0 Array [CDF: Mm_ENTREZG_10] | 2011 | USA | (Bethunaickan et al., 2011) |
| GSE32591 | Human | NA | Kidney (tubulointerstitial and glomeruli compartments) | RNA from glomeruli and tubulointerstitial compartments was extracted and processed for hybridization on Affymetrix microarrays. | (1) Nephritis (n=32 for renal tubulointerstitial biopsies, GSM807842-GSM897873; n=32 for renal glomeruli biopsies, GSM807889-GSM807920)  (2) Health control (n=15 for renal tubulointerstitial biopsies, GSM807874-GSM807888; n=14 for renal glomeruli biopsies, GSM807921-GSM807934) | GPL14663, Affymetrix GeneChip Human Genome HG-U133A Custom CDF [Affy_HGU133A_CDF_ENTREZG_10] | 2012 | USA | (Berthier et al., 2012) |

References:

Berthier, C.C., Bethunaickan, R., Gonzalez-Rivera, T., Nair, V., Ramanujam, M., Zhang, W., Bottinger, E.P., Segerer, S., Lindenmeyer, M., Cohen, C.D.*, et al.* (2012). Cross-species transcriptional network analysis defines shared inflammatory responses in murine and human lupus nephritis. J Immunol *189*, 988-1001.

Bethunaickan, R., Berthier, C.C., Ramanujam, M., Sahu, R., Zhang, W., Sun, Y., Bottinger, E.P., Ivashkiv, L., Kretzler, M., and Davidson, A. (2011). A unique hybrid renal mononuclear phagocyte activation phenotype in murine systemic lupus erythematosus nephritis. J Immunol *186*, 4994-5003.

Bethunaickan, R., Berthier, C.C., Zhang, W., Eksi, R., Li, H.D., Guan, Y., Kretzler, M., and Davidson, A. (2014). Identification of stage-specific genes associated with lupus nephritis and response to remission induction in (NZB x NZW)F1 and NZM2410 mice. Arthritis Rheumatol *66*, 2246-2258.

Schiffer, L., Sinha, J., Wang, X., Huang, W., von Gersdorff, G., Schiffer, M., Madaio, M.P., and Davidson, A. (2003). Short term administration of costimulatory blockade and cyclophosphamide induces remission of systemic lupus erythematosus nephritis in NZB/W F1 mice by a mechanism downstream of renal immune complex deposition. J Immunol *171*, 489-497.
