# Supplementary material for: Immune Cell Landscape Identification Associates Intrarenal Mononuclear Phagocytes With Onset and Remission of Lupus Nephritis in NZB/W Mice
Source: Front Genet. 2020 Nov 9;11:577040. doi: 10.3389/fgene.2020.577040 (PMC7693546; doi:10.3389/fgene.2020.577040)
Supplement: Supplementary Table 5 — LN onset-related hub genes identified from DEGs overlapped between GSE27045 and GSE32583. [file Table_5.DOCX]

**Supplementary Table S5 ∣ LN onset-related hub genes identified from overplapped DEGs between GSE27045 and GSE32583.**

|  | **Pre-nephritis vs. Nephritis (GSE27045)** | | **Pre-nephritis vs. Nephritis (GSE32583)** | |
| --- | --- | --- | --- | --- |
| **Mouse Gene Symbol** | **LogFC** | **Adjusted p-value** | **LogFC** | **Adjusted p-value** |
| Myd88 | 0.56943070 | 0.00253175 | 0.40100000 | 0.00000623 |
| Lyn | 0.43167200 | 0.00070394 | 0.58500000 | 0.00000674 |
| C3 | 1.22406010 | 0.00011052 | 1.50000000 | 0.00000478 |
| Vcam1 | 1.38554710 | 0.00008783 | 1.07000000 | 0.00000898 |
| Rac2 | 0.51502430 | 0.00477454 | 0.52700000 | 0.00020300 |
| Fpr2 | 1.16986820 | 0.00004614 | 1.81000000 | 0.00000205 |
| Cd274 | 0.98857120 | 0.01130228 | 0.37700000 | 0.00775000 |
| Cd44 | 0.73371410 | 0.00294004 | 1.61000000 | 0.00000024 |
| Cd28 | -1.75804110 | 0.00022277 | 0.33700000 | 0.00147000 |
| Fcgr4 | 0.56581860 | 0.00028784 | 1.28000000 | 0.00005210 |
| Il10 | 2.46665750 | 0.01096820 | 0.16300000 | 0.00314000 |
| Fcgr1 | 0.63254540 | 0.00605082 | 0.57500000 | 0.00000303 |
| Stat3 | 0.63575200 | 0.00019961 | 0.63200000 | 0.00000120 |
| Itgb2 | 0.38249990 | 0.00924515 | 1.44000000 | 0.00000075 |
| Ccl2 | 1.21107310 | 0.00385386 | 0.56300000 | 0.00038100 |
| Itgam | 2.09038430 | 0.00000879 | 0.94300000 | 0.00001180 |
| Lcp2 | 0.73400610 | 0.00027466 | 0.36600000 | 0.00006010 |
| Cd40 | 1.19334520 | 0.00035172 | 0.27300000 | 0.00094000 |
| Cxcr4 | 0.66461590 | 0.00291133 | 0.89600000 | 0.00000624 |
| Fyn | 0.87306850 | 0.00047549 | 0.44000000 | 0.00002780 |

LN, lupus nephritis; LogFC, log2 ^fold change^.
