# Supplementary material for: Immune Cell Landscape Identification Associates Intrarenal Mononuclear Phagocytes With Onset and Remission of Lupus Nephritis in NZB/W Mice
Source: Front Genet. 2020 Nov 9;11:577040. doi: 10.3389/fgene.2020.577040 (PMC7693546; doi:10.3389/fgene.2020.577040)
Supplement: Supplementary Table 7 — LN remission-related hub genes in GSE27045. [file Table_7.DOCX]

**Supplementary Table S7 ∣ LN remission-related hub genes in GSE27045.**

|  | **Nephritis vs. Remission** | |
| --- | --- | --- |
| **Mouse Gene Symbol** | **LogFC** | **Adjusted p-value** |
| Aurkb | -1.2949702 | 0.00039 |
| Mki67 | -1.0460513 | 0.012013 |
| Nusap1 | -1.3246615 | 0.000844 |
| Cdca8 | -1.0812838 | 0.000571 |
| Birc5 | -1.4906916 | 0.000186 |
| Prc1 | -1.1576969 | 0.004167 |
| Ccna2 | -1.2220427 | 0.001379 |
| Pbk | -1.2085621 | 0.007573 |
| Cdk1 | -1.0474923 | 0.00425 |
| Kif4 | -1.2579106 | 0.000194 |
| Bub1b | -1.367622 | 0.001352 |
| Cdca5 | -1.2216232 | 0.00039 |
| Kif11 | -1.1123784 | 0.000921 |
| Nuf2 | -1.3836537 | 0.001189 |
| Ccnb1 | -1.6032193 | 0.00184 |
| Ccnb2 | -1.5574987 | 0.000125 |
| Plk1 | -1.1896002 | 0.001379 |
| Bub1 | -1.367622 | 0.001352 |
| Kif20a | -1.0894058 | 0.007333 |
| Cdc20 | -1.1161544 | 0.000844 |

LN, lupus nephritis; LogFC, log2 ^fold change^.
