# Supplementary material for: Immune Cell Landscape Identification Associates Intrarenal Mononuclear Phagocytes With Onset and Remission of Lupus Nephritis in NZB/W Mice
Source: Front Genet. 2020 Nov 9;11:577040. doi: 10.3389/fgene.2020.577040 (PMC7693546; doi:10.3389/fgene.2020.577040)
Supplement: Supplementary Table 8 — Validation of LN onset-related hub genes in GSE32591. [file Table_8.DOCX]

**Supplementary Table S8 ∣ Validation of LN onset-related hub genes in GSE32591.**

|  | **LD vs. LN (Tubulointerstitial compartment)** | | **LD vs. LN (Glomerular compartment)** | |
| --- | --- | --- | --- | --- |
| **Human Gene Symbol** | **LogFC** | **Adjusted p-value** | **LogFC** | **Adjusted p-value** |
| MYD88 | 0.28561254 | 3.40E-03 | 1.078805 | 8.63E-09 |
| LYN | 0.62162926 | 1.12E-04 | 2.2191317 | 1.14E-09 |
| C3 | 0.75930927 | 3.55E-02 | 0.8391333 | 8.87E-02 |
| VCAM1 | 0.97088698 | 6.93E-04 | 0.3785939 | 1.11E-01 |
| RAC2 | 0.42488475 | 2.62E-02 | 1.4023359 | 4.15E-06 |
| FPR2 | -0.19388462 | 1.15E-04 | 0.4009159 | 1.97E-02 |
| CD44 | 0.27278726 | 1.79E-02 | 1.1977428 | 3.21E-05 |
| CD28 | -0.04540486 | 2.87E-01 | 0.0197367 | 7.14E-01 |
| FCGR3B | 0.00500913 | 9.76E-01 | 1.5547608 | 8.82E-04 |
| IL10 | -0.12371545 | 1.51E-02 | 0.0410356 | 4.57E-01 |
| STAT3 | 0.23890695 | 5.34E-02 | 0.0729495 | 7.32E-01 |
| ITGB2 | 0.86742064 | 4.81E-04 | 2.6929469 | 8.00E-09 |
| CCL2 | -0.31855878 | 4.04E-01 | 0.3784334 | 4.36E-01 |
| ITGAM | 0.11668845 | 1.77E-01 | 1.3790114 | 1.08E-04 |
| LCP2 | 0.18065972 | 2.38E-02 | 1.2443814 | 2.24E-06 |
| CD40 | 0.07823106 | 2.43E-01 | 0.4304905 | 1.07E-03 |
| CXCR4 | 0.64444957 | 1.14E-02 | 1.6153557 | 9.54E-05 |
| FYN | 0.18039976 | 2.46E-01 | -0.5499227 | 1.12E-03 |

LN, lupus nephritis; LD, healthy living control; LogFC, log2 ^fold change^.
